# Bibliometrics and scientometrics analysis of exosomes relevance in hepatocellular carcinoma (2014-2024)

**DOI:** 10.3389/fonc.2025.1614484

**Published:** 2025-09-16

**Authors:** Kaikui Wu, Guiya Lu, Ruijun Guo, Chunxia Li, Minglin Ou

**Affiliations:** ^1^ Laboratory Center, Guangxi Key Laboratory of Metabolic Reprogramming and Intelligent Medical Engineering for Chronic Diseases, The Second Affiliated Hospital of Guilin Medical University, Guilin, China; ^2^ Laboratory Center, Guangxi Health Commission Key Laboratory of Glucose and Lipid Metabolism Disorders, The Second Affiliated Hospital of Guilin Medical University, Guilin, China; ^3^ School of Life Sciences, Guangxi Normal University, Guilin, China

**Keywords:** exosomes, hepatocellular carcinoma, bibliometric analysis, research trends, hotspot

## Abstract

**Background:**

Exosomes have emerged as pivotal players in the pathogenesis, progression, and treatment of hepatocellular carcinoma (HCC), establishing them as a major research focus in recent years. This bibliometric analysis maps the knowledge evolution and quantifies research trends in exosome-associated HCC studies from 2014 to 2024.

**Methods:**

We retrieved relevant publications (2014-2024) from the Web of Science Core Collection (WoSCC) database and conducted bibliometric analyses using CiteSpace, VOSviewer, and R software.

**Results:**

The analysis encompassed 1,120 publications (714 research articles and 406 reviews), originating from 60 countries/regions and 4,035 institutions, published in 388 journals, and authored by 6,989 authors. China emerged as the most productive country, followed by the United States. The most prolific journals were the International Journal of Molecular Sciences and Frontiers in Oncology, while Fudan University ranked as the most productive institution. Trend analysis indicates that future HCC exosome research will concentrate on: the development and application of exosomes as HCC biomarkers, optimizing exosomes as drug delivery vehicles, investigating tumor treatment resistance, and exploring exosome-mediated immunotherapeutic strategies for HCC to facilitate their clinical application.

**Conclusion:**

Current evidence demonstrates that the field of HCC exosome research is in a phase of rapid development, with its potential in both basic research and clinical translation continually being explored. Through bibliometric analysis, this study identifies key trends and emerging directions in HCC exosome research from 2014 to 2024, offering insights into current research priorities and potential innovations.

## 1 Introduction

Cancer remains a growing global health challenge, with incidence rates steadily increasing worldwide. HCC is the predominant type of liver cancer (1). As of the 2024 global cancer statistics, the survival rate among individuals with HCC stands at a mere 22% (2), thus posing a significant threat to public health. Therefore, preventing, controlling, and treating HCC pose a major challenge to global public health.

HCC is characterized by insidious early-stage symptoms, with most patients diagnosed at an advanced stage, resulting in limited treatment options and poor prognosis (3). Clinical interventions include resection, transplantation, ablation, transarterial therapy, radiotherapy, and systemic therapy (4). The emergence of immune checkpoint inhibitors (ICIs) marked a milestone in HCC treatment, significantly transforming the therapeutic landscape for advanced HCC (5). Fu Y et al. proposed that ICI-based combination therapies can significantly improve survival benefits in patients with advanced HCC compared to sorafenib monotherapy, establishing this approach as the new standard for first-line treatment (6). In this context, a study by Wen F et al. indicates that for patients without contraindications, camrelizumab combined with rivoceranib may represent a highly cost-effective strategy (7).Targeted therapy has also advanced. This includes novel delivery systems such as the metal-organic framework (MOF)-based platform developed by Huang et al., which effectively transports chemotherapeutic agents, demonstrating potent antitumor activity with reduced side effects (8). More broadly, targeted drugs offer enhanced safety and specificity through selectively acting on tumor signaling pathways (9). Lenvatinib, a multi-target tyrosine kinase inhibitor, has emerged as an effective first-line alternative to sorafenib. Crucially, its combination with immunotherapy shows promise in delaying the onset of drug resistance or overcoming resistance (10). However, both targeted therapy and chemotherapy are significantly limited by primary or acquired drug resistance, which ultimately leads to disease recurrence. Sorafenib is constrained by limited efficacy and significant drug resistance issues, while chemotherapy is hindered by multidrug resistance mechanisms. Therefore, developing and applying innovative combination strategies involving two or more therapeutic agents represents a core strategy for overcoming HCC drug resistance and improving clinical efficacy (11). Additionally, the lack of reliable predictive biomarkers hinders the achievement of precision medicine and the optimization of patient selection (12).

To address these challenges, exosomes have garnered significant attention because of their critical pathophysiological roles in tumor progression. Exosomes are a specific subtype of extracellular vesicles, typically with a diameter ranging from 30 to 150 nm. Encased in a lipid bilayer membrane, they carry diverse molecular cargo—including DNA, RNA, proteins, and lipids—that mediate intercellular communication and regulate key pathophysiological processes (13, 14). In HCC, studies have demonstrated that exosomes promote tumor growth and metastasis by delivering bioactive molecules, playing a critical role in its development and progression (15, 16). Crucially, exosomes exhibit substantial potential in early diagnosis, prognostic assessment, and therapeutic efficacy monitoring for HCC, offering novel insights for advancing precision medicine (17, 18). Given their significant roles, research into exosomes in HCC has expanded rapidly worldwide. Scientists have intensively investigated their biological functions, uncovering essential mechanisms underlying hepatocarcinogenesis, as well as progression, proliferation, migration, invasion, and immune escape (19, 20). Concurrently, as key components of liquid biopsies, exosomes demonstrate considerable clinical value in early diagnosis, prognostic evaluation, and treatment response monitoring for HCC, attributable to their unique advantages: non-invasiveness, the capacity for comprehensive tumor molecular profiling, high stability, and ease of detection (21, 22). Nevertheless, despite notable advances, the field faces significant challenges. Current exosome isolation techniques are often limited by low efficiency and insufficient yield, severely constraining their large-scale clinical translation and posing significant obstacles (23).

Amid the rapid advancement of science and technology, researchers encounter two major challenges: identifying innovative projects among a vast array of research topics, and integrating findings across a growing number of disciplines. In the face of the ever-growing volume of literature and research results, bibliometric analysis has become an important tool. It offers researchers an efficient approach to understand new areas by analyzing publication volume within a specific field. This method not only facilitates the examination of historical achievements but also identifies under-explored research frontiers. Importantly, despite remarkable progress and a substantial accumulation of research findings on exosomes in HCC, a comprehensive quantitative analysis and systematic evaluation of the current research landscape is still lacking. Utilizing bibliometric methods, this study conducts a systematic analysis of global trends and research hotspots in exosome applications for HCC. It aims to offer researchers a comprehensive overview of the current state of research and to outline potential future directions and applications. Based on the analysis presented in this study, we aim to provide valuable insights to support the further development and clinical translation of HCC exosome research.

## 2 Materials and methods

### 2.1 Data sources and search strategies

**Figure 1:**
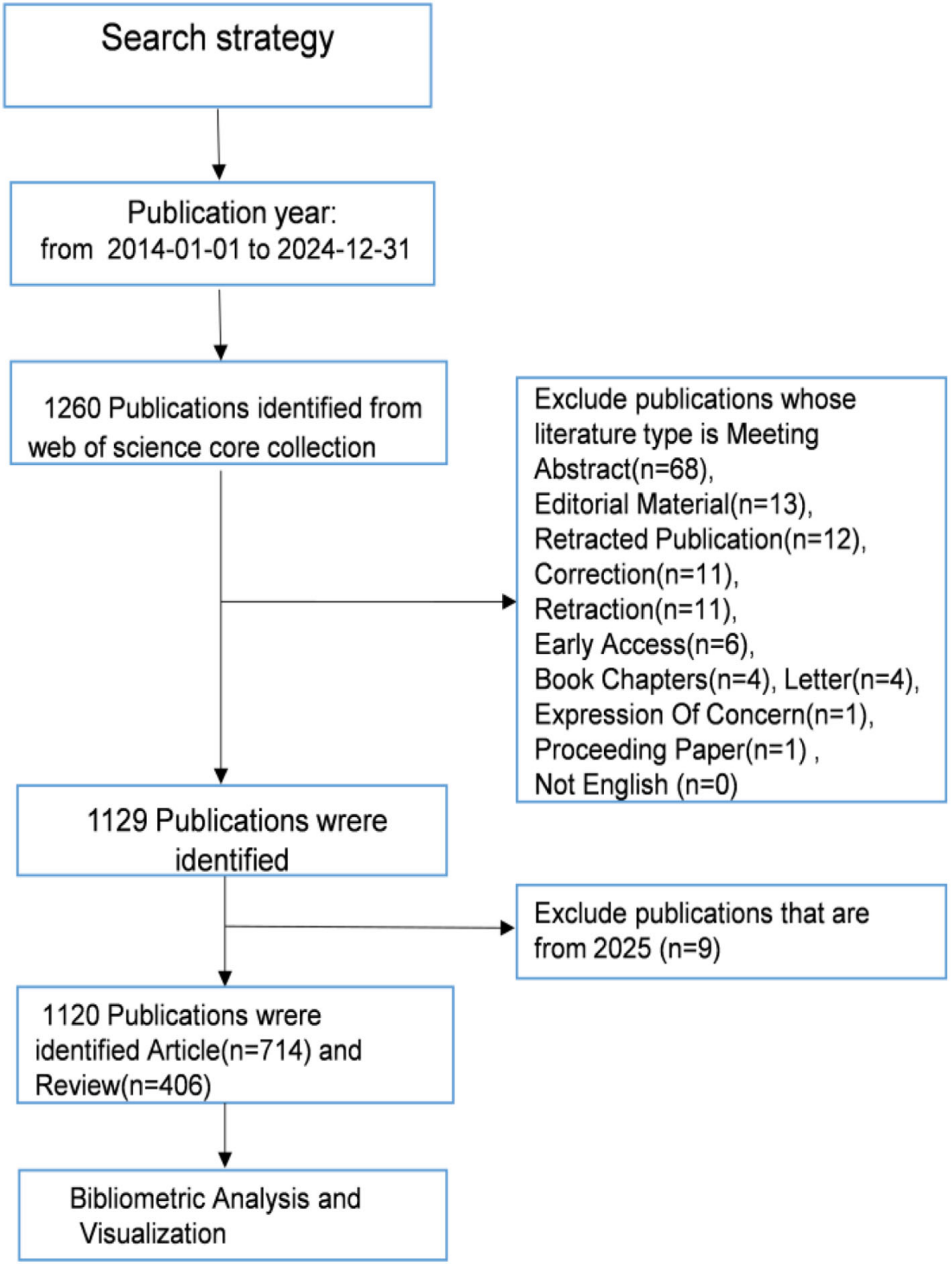
Literature retrieval flowchart.

The Web of Science Core Collection (WoSCC), a globally recognized citation database, serves as a critical resource for scholarly research and bibliometric studies by providing authoritative literature coverage. In this study, a comprehensive literature search of the WoSCC database was conducted on February 26, 2025, with the following search strategy: TS=((“hepatocellular carcinom*” OR “hepatocellular cancer*” OR “hepatic tumor*” OR “hepatic tumour*” OR “primary liver cancer*” OR HCC)) AND TS= ((“exosom*”OR”exosomal cargo” OR”exosome-derived” OR”exosome-mediated”) NOT (“microvesicle*” OR”ectosome*”OR”apoptotic bod*” OR”large oncosom*”)). To ensure the accuracy and objectivity of the analysis, only articles and reviews were selected, while conference abstracts, editorials, book chapters, and conference proceedings were excluded. The search was restricted to publications published between January 1, 2014, and December 31, 2024, and only English-language articles and reviews relevant to the topic were included. After initial retrieval and screening, a final set of 1,120 publications was exported in both “Full Record and Cited References” and “Plain Text” file formats for subsequent data processing and analysis. The process of literature search and screening is depicted in **Figure 1**.

### 2.2 Data analysis

In this study, we employed several software tools to conduct comprehensive bibliometric analyses: R, VOSviewer, CiteSpace, and Excel. VOSviewer, which specializes in constructing and visualizing scientific knowledge graphs (24), was employed to examine collaborations among countries, authors, and co-citation patterns of authors and journals. CiteSpace, which tracks the dynamic evolution of research hotspots through burst intensity and duration metrics (25), was used for burst analyses of keywords and references, journal dual-map overlay analysis, keyword co-occurrence and timeline graph analysis, as well as institutional analysis, and centrality analysis of authors and co-cited authors. Additionally, Excel was utilized to analyze publication volume and trends, while the ggplot2 package in R was employed to generate bar cand scatter plots.

## 3 Results

### 3.1 Analysis of publication trends

**Figure 2:**
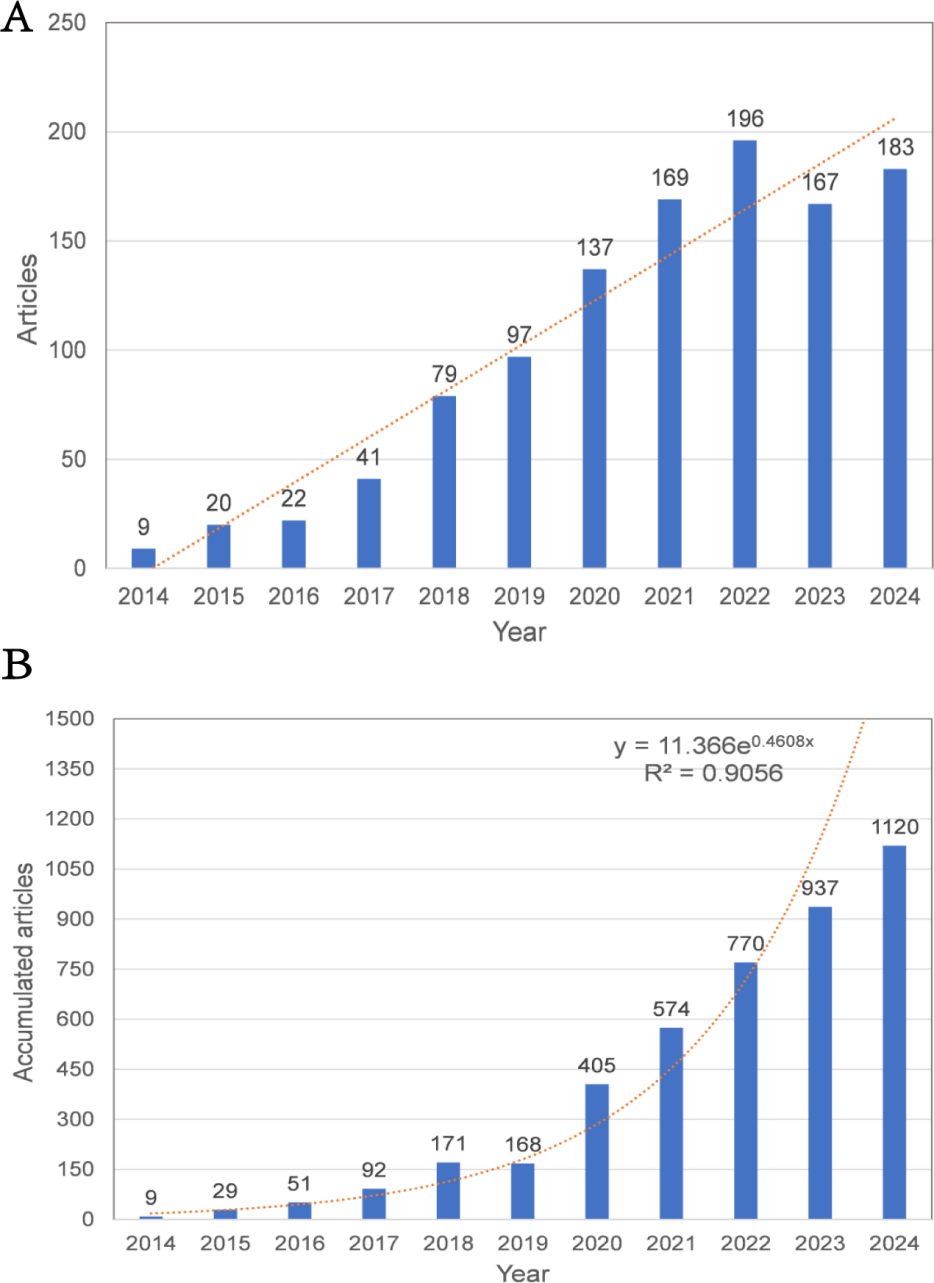
**(A)** Annual publication volume and trend line. **(B)** Collaboration publication volume and trend line.

Between 2014 and 2024, a total of 1,120 publications related to HCC exosomes were included in this study. **Figure 2** illustrates the annual publication volume and the cumulative number of publications during this period. The initial publication count was 9 in 2014, followed by a steady annual increase. Notably, a significant surge occurred in 2018, with the number of articles jumping from 41 in 2017 to 79. The peak was reached in 2022, with 196 articles published. Although there was a slight decline in annual publications in 2023 and 2024, the cumulative trajectory continues its upward trend, reflecting sustained academic interest in this field. Furthermore, by 2024, the cumulative publication output exceeded 1,000 articles, demonstrating rapid growth overall.

### 3.2 Analysis of the number of national publications

A global evaluation of academic productivity identified scholarly contributions from 60 countries/regions in HCC exosome research. According to **Table 1**, China dominated the field with 772 publications and 35,861 total citations, followed by the United States with 138 publications and 11,747 citations. The remaining countries/regions each published fewer than 50 papers. This distribution highlights China and the United States as the most significant contributors with the greatest influence in this research domain. When considering average citations per article, the United States ranked first at 85.12 citations per paper. Other nations demonstrating strong academic influence included Iran (54.22), Japan (53.51), and Germany (45.99). This metric suggests that despite lower publication volumes than China, these countries produce scholarly work of exceptional quality and scientific impact. Such high-impact research reflects not only substantial research infrastructure and rigorous academic standards in these countries, but also their capacity for scientific innovation.

**Table 1:** Global research output on HCC exosomes: top 10 contributing countries/regions.

Rank	Country/region	Publications	Citations	Average citations	Total link strength
1	China	772	35861	46.45	101
2	USA	138	11747	85.12	127
3	Italy	49	1868	38.12	41
4	Iran	45	2440	54.22	56
5	Japan	45	2408	53.51	17
6	Germany	39	1687	45.59	48
7	India	32	841	26.28	38
8	South Korea	27	1140	42.22	4
9	Egypt	19	552	29.05	17
10	Spain	18	696	38.67	25

**Figure 3:**
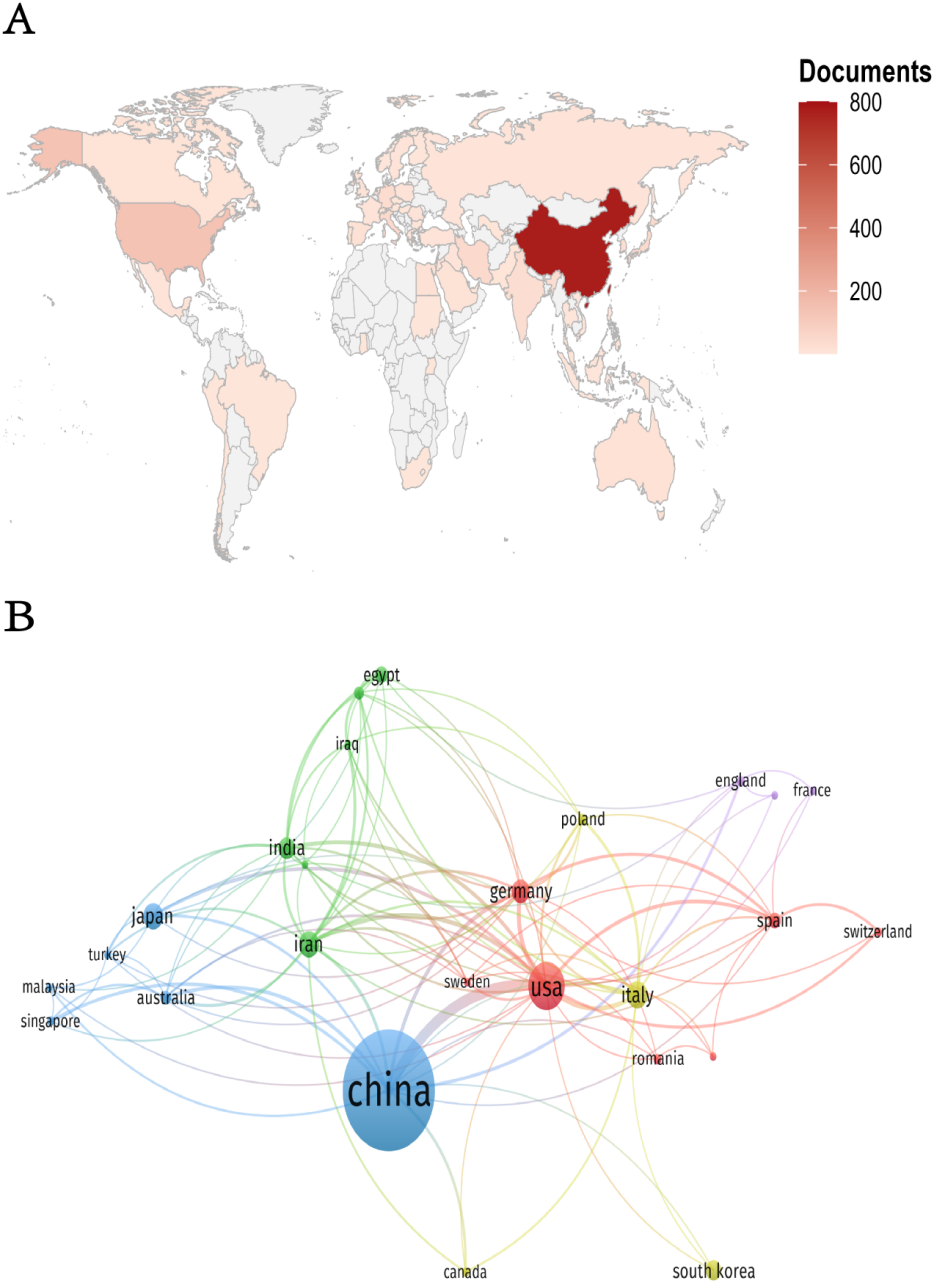
**(A)** Geographic distribution map of countries/regions. **(B)** Collaboration network diagram of countries/regions.

**Figure 3** visualizes international collaboration patterns. **Figure 3A** displays a country/region visualization map where color intensity corresponds to publication volume (darker shades = higher output; gray = no publications). **Figure 3B** illustrates co-occurrence networks among 25 countries/regions, with the United States and China as core hubs. The thickest connections between these two nations indicate their strongest collaborative ties. Both countries maintain dense connections with multiple partners, underscoring their centrality in global research networks.

### 3.3 Analysis of institutional outputs and cooperation

**Figure 4:**
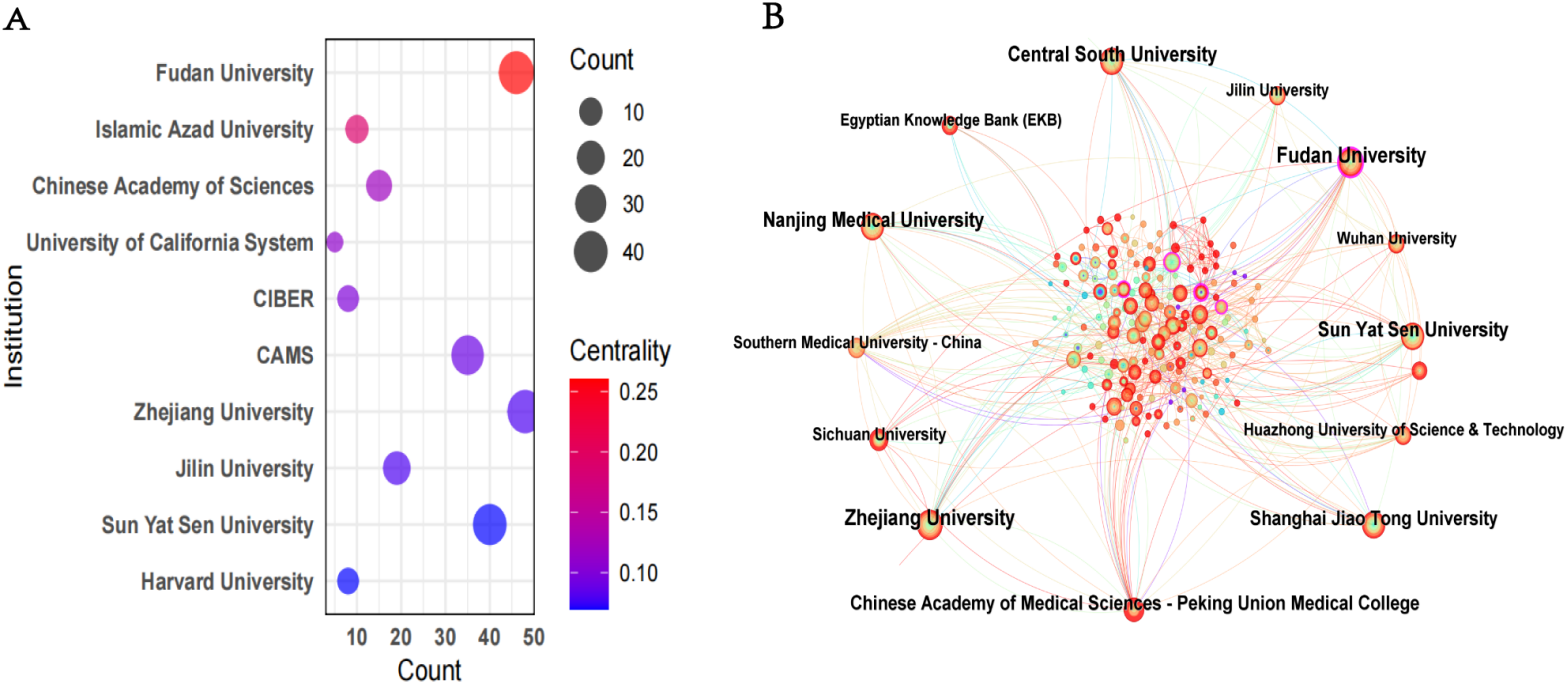
**(A)** The ten institutions with the highest centrality. The full name of CAMS is “Chinese Academy of Medical Sciences”. The full name of CIBER is “Centro de Investigación Biomédica en Red”. **(B)** Network map of institutional collaborations.

The study identified 4,035 organisations involved in research into HCC exosomes. The following **Table 2** presents the top 11 most productive organisations in this field. Notably, all 11 institutions with the highest output are from China, highlighting China’s dominance in HCC exosome research. Zhejiang University led in publication volume with 48 articles, followed closely by Fudan University (46 articles), Sun Yat-sen University (40 articles), and Nanjing Medical University (39 articles), highlighting their substantial contributions to scholarly output in HCC exosome studies. In terms of citation impact, Fudan University achieved the highest total citations (2,978), marginally surpassing Zhejiang University (2,911) and Sun Yat-sen University (2,652). Notably, Shanghai Jiao Tong University demonstrated exceptional research influence, attaining 2,392 citations from only 33 publications – the highest average citation rate (72.48 per article) among ranked institutions. This metric underscores the institution’s capacity to produce high-impact studies despite a comparatively lower publication volume. Additionally, institutions such as Sichuan University, Southern Medical University, Wuhan University, Huazhong University of Science and Technology, and China Medical University have also made substantial research contributions, further solidifying China’s comprehensive leadership in HCC exosome studies. In terms of centrality (**Figure 4A**), Fudan University had the highest score of 0.26, followed by the Islamic Azad University with 0.19. Additionally, we used CiteSpace to map the collaboration network among institutions (**Figure 4B**). In this visualization, the size of the nodes represents the number of articles published by the respective institutions, the connecting lines between nodes indicate collaborative relationships, and the thickness of the lines reflects the intensity or frequency of cooperation. Fudan University and Shanghai Jiao Tong University stand out in this collaboration network. They not only have a relatively large number of published papers but also maintain extensive and close collaborations with other institutions. As core institutions with significant influence in this research field, they play a key role in promoting inter-institutional cooperation and research progress.

**Table 2:** Top 11 manufacturing organizations in the field of HCC exosome.

Rank	Organization	Country	Count	Citations	Average citations
1	Zhejiang univ	China	48	2911	60.65
2	Fudan univ	China	46	2978	64.74
3	Sun yat sen univ	China	40	2652	66.30
4	Nanjing med univ	China	39	2405	61.67
5	Cent south univ	China	38	1245	32.76
6	Shanghai jiao tong univ	China	33	2392	72.48
7	Sichuan univ	China	23	1104	47.96
8	Southern med univ	China	20	632	31.60
9	Wuhan univ	China	20	421	21.05
10	Hua zhong univ sci & technol	China	19	609	32.05
11	China med univ	China	19	955	50.26

### 3.4 Journal analysis

**Figure 5:**
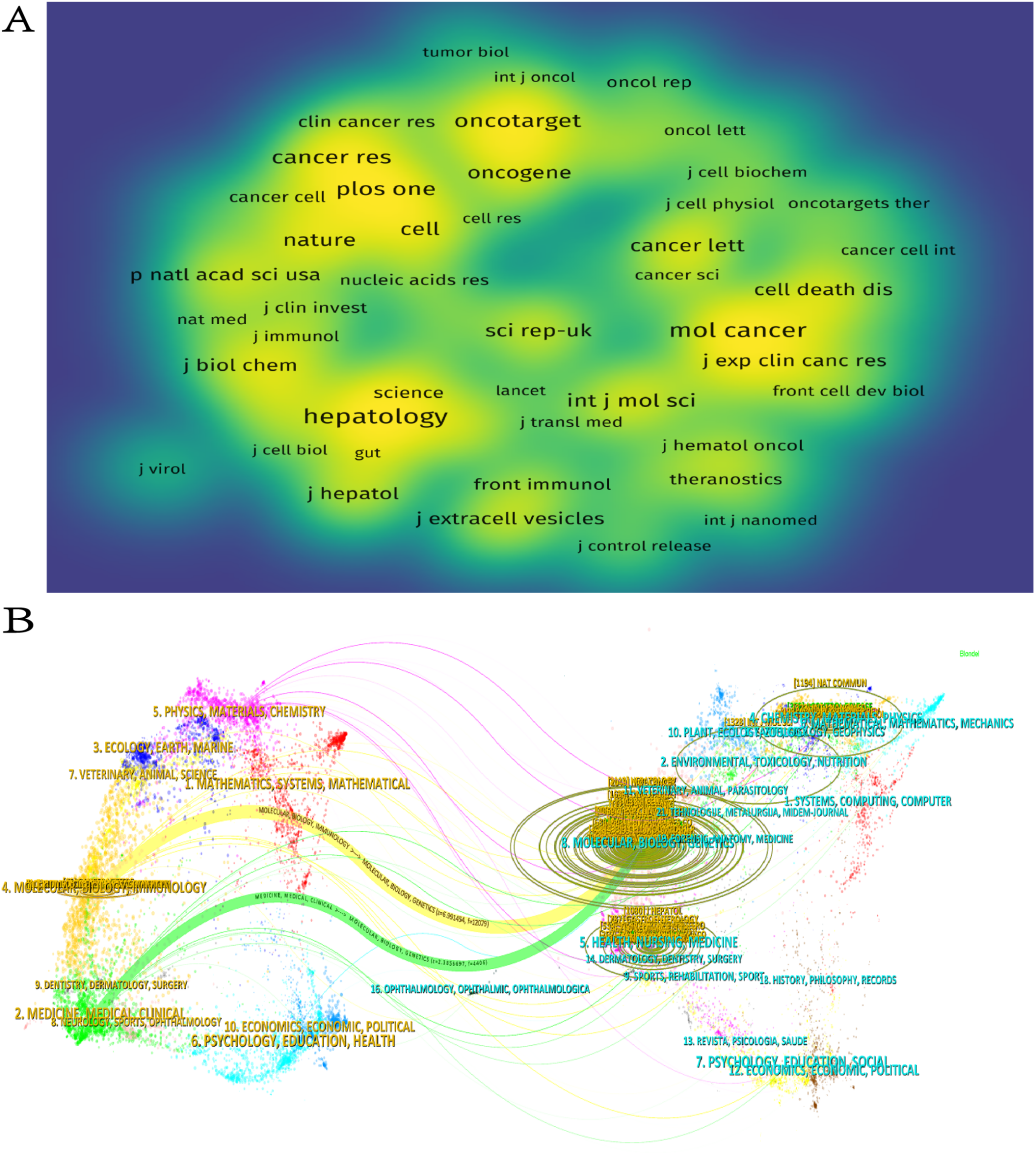
**(A)** Journal density maps constructed on the basis of co-citations. **(B)** Journal double figures.

The study revealed that 388 distinct journals featured 1,120 articles associated with HCC exosomes. lists the ten most productive journals in this field. Collectively,according to **Table 3**, these top 10 journals published 210 papers, with the International Journal of Molecular Sciences leading (n=37), followed by Frontiers in Oncology (n=33), Cancers (n=28), and Molecular Cancer (n=19). Switzerland dominates among high-productivity journals, with three of the top 10 journals originating there. Notably, no Chinese journals appear on this list, indicating that China’s research output has not yet translated into journal platform influence. **Figure 5A** visualizes journal co-citation networks. Warmer colors (red spectrum) denote higher co-citation frequency, highlighting influential journals like Hepatology, Molecular Cancer, and Oncotarget. **Figure 5B** presents a dual-map overlay of citation flows, where the thickest green and yellow pathways indicate dominant knowledge transfer: publications in Molecular/Biology/Immunology and Medicine/Medical/Clinical domains are predominantly cited by Molecular/Biology/Genetics and Health/Nursing/Medicine research.

**Table 3:** Top 10 most productive journals in HCC exosome research.

Rank	Source journals	Articles	Country	Citations	Average citations
1	International Journal of Molecular Sciences	37	Switzerland	1480	40.00
2	Rontiers in Oncology	33	Switzerland	403	12.21
3	Cancers	28	Switzerland	604	21.57
4	Molecular Cancer	19	UK	4308	226.74
5	Frontiers in Cell and Developmental Biology	18	Switzerland	504	28.00
6	Cells	18	Switzerland	744	41.22
7	Cancer Letters	16	Netherlands	859	53.69
8	Cell Death & Disease	15	Switzerland	773	51.53
9	Scientific Reports	14	UK/Germany	503	35.93
10	Hepatology	12	US	1533	127.75

### 3.5 Analysis of authors and co-cited authors

**Figure 6:**
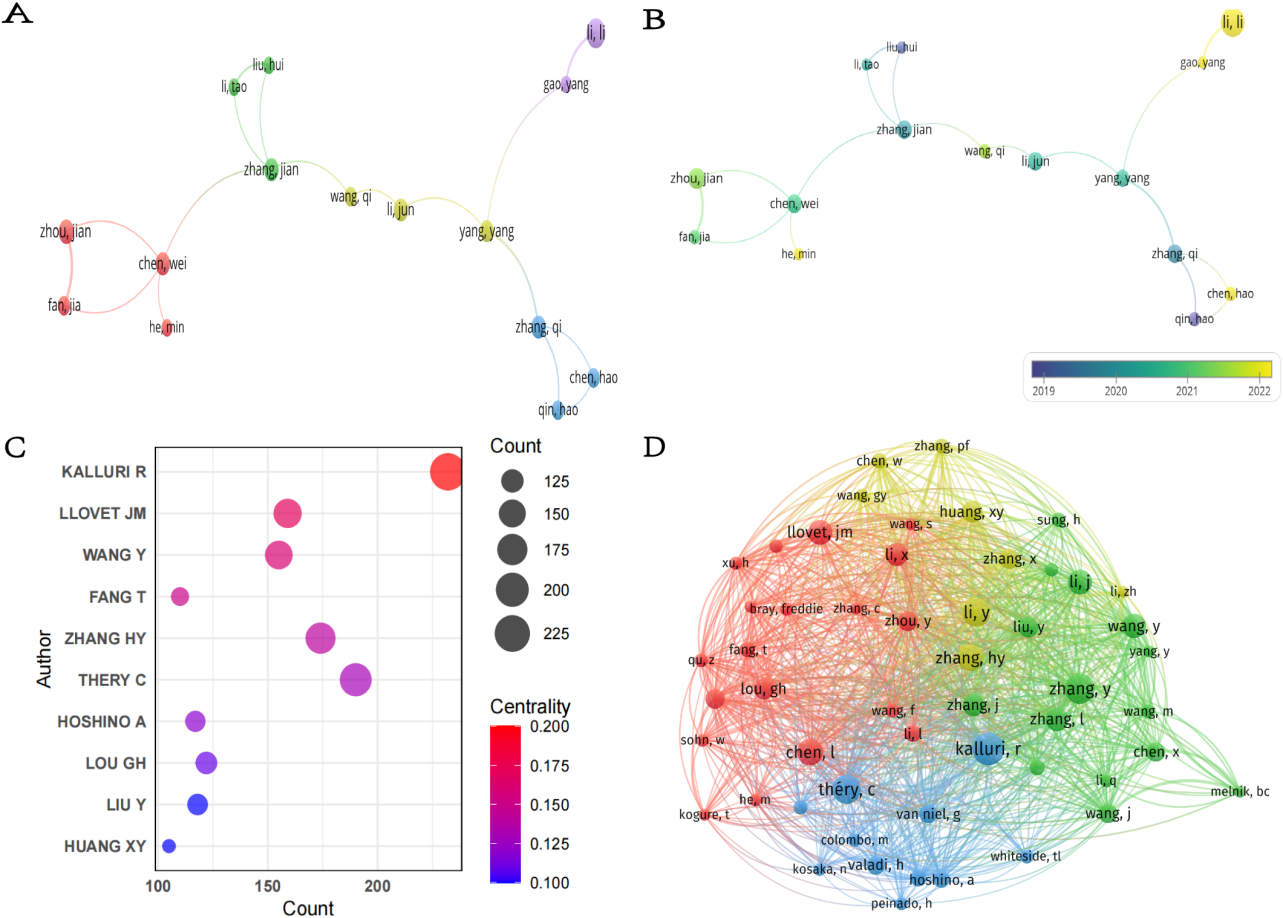
**(A)** Collaboration network of authors. **(B)** Temporal overlay map of co-authorship analysis. **(C)** Top seven co-cited authors with the highest centrality. **(D)** Co-citation network of authors.

Overall,6,989 authors participated in the publication of these 1,120 papers. In terms of publication output, Li L was the most prolific author with 13 papers (**Table 4**), followed by Zhou J and Patel T, each with 9 papers. Regarding citation impact, Patel T had the highest aggregate citation total, accumulating 1,009 citations, followed by Zhang Q with 707 citations. The highest average citation count per article was achieved/attained by Zhou J (112.11), followed by Zhang Q (88.38) and Yam JWP (82.13).These figures highlight the significant influence of these authors within the field. It is significant to acknowledge that the centrality of all ten of these prolific authors is 0.00, indicating that their collaborations may be focused within a fixed team or institution and lack cross-network collaborations with other teams. In the future, it is necessary to enhance their structural influence by collaborating with teams from different countries or institutions to promote collaborative innovation in the field of HCC exosomes. As shown in **Figure 6A**, 16 researchers were categorized into four clusters, which were led by Li L, Zhang Q, Zhang J, Chen W and Yang Y respectively. Furthermore, there is cooperation among different clusters. Fan J, despite having only 6 articles, is currently pursuing opportunities for collaborative projects with other authors. In addition, the node colors in **Figure 6B** provide temporal information, transitioning from purple (2019) to yellow (2022), reflecting the authors’ activity over different years. For example, the yellow nodes of researchers such as Li L, Gao Y, Chen H, and He M indicate their active involvement in new research within the field of HCC exosomes.

**Table 4:** Ten authors with the highest number of publications in HCC.

Rank	Author	Publication	Citations	Average citations	Centrality
1	Li L	13	335	25.77	0.00
2	Patel T	9	653	72.56	0.00
3	Zhou J	9	1009	112.11	0.00
4	Zhang Q	8	707	88.38	0.00
5	Wang Y	8	397	49.63	0.00
6	Xu Y	8	505	63.13	0.00
7	Yam JWP	8	657	82.13	0.00
8	Zhang J	8	242	30.25	0.00
9	Liu H	7	116	16.57	0.00
10	Yang Y	7	492	70.29	0.00

In the academic literature, a co-citation relationship is formed when two or more authors’ research is cited together in the same document (26). **Figure 6C** shows the top ten co-cited authors with the highest centrality in the field of HCC exosome research, along with their co-citation frequency and centrality metrics. The chart details each author’s influence and centrality in the academic network. The most cited author is Kalluri R (232), followed by Thery C (190) and Zhang HY (174).These authors are highly influential in the academic community. Kalluri R also ranked first with a centrality index of 0.20, indicating that this author holds a central position in the field, and Llovet JM ranked second with 0.17. Notably, although Thery C has the second highest co-citation frequency, her centrality (0.13) is relatively low, suggesting that her research may be more focused on specific niches/subfields/directions. Furthermore, using VOSviewer (**Figure 6D**), we visualized the co-citation network of authors. Authors such as Kalluri R, Chen L, Zhang HY, and Thery C not only have high co-citation frequencies but also form a closely interconnected academic network with several high-impact scholars.

### 3.6 The 10 most cited publications

Highly cited publications serve as critical indicators of academic influence and hold substantial reference value in scholarly studies. Through systematic analysis of impactful literature, researchers can not only identify emerging trends within specialized domains but also establish theoretical foundations for subsequent in-depth studies that build upon existing findings (27). There are a total of 59,753 references in articles in the field of HCC exosomes. Using bibliometrix, we statistically analyzed the top ten publications in terms of citations (**Table 5**) and found that all ten were published between 2015 and 2020, with more than half cited more than 440 times. The most cited article is “Targeting Cancer Stem Cell Pathways for Cancer Therapy”, published by Yang, LQ et al. in “Signal Transduct Target Ther” in 2020, with an average of 194.50 annual citations (28). The second most cited publication is “Exosomes: Composition, Biogenesis, and Mechanisms in Cancer Metastasis and Drug Resistance”, authored by Mashouri, L et al., published in Molecular Cancer in 2019, with an average of 149.14 citations per year (29). Collectively, these influential articles comprehensively address both fundamental mechanisms and clinical applications of exosomes in HCC research.

**Table 5:** The top 10 cited publications.

Rank	Title	Year, Journal	First author	Total Citations	TC per Year
1	Targeting cancer stem cell pathwaysfor cancer therapy	2020, Signal Transmission TAR	YANG LQ	1132	194.50
2	Exosomes: composition, biogenesis, and mechanisms in cancer metastasis and drug resistance	2019, MOL CANCER	MASHOURI L	1044	149.14
3	Tumor-derivedexosomal miR-1247-3p induces cancer-associated fibroblast activation to foster lung metastasis of hepatocellular	2018, NAT COMMUN	FANG T	771	96.38
4	Exosomes derived from miR-122-modified adipose tissue-derived MSCs increase chemosensitivity of hepatocellular carcinoma	2015, J HEMATOL ONCOL	LOU GH	570	51.82
5	Carcinoma-associated fibroblasts promote the stemness and chemoresistance of colorectal cancer by transferring exosomal lncRNA H19	2018, THERANOSTICS	REN J	548	68.50
6	The significance of exosomes in the development and treatment of hepatocellular carcinoma	2020, MOL CANCER	LI X	440	73.33
7	Comprehensive toxicity and immunogenicity studies reveal minimal effects in mice following sustained dosing of extracellular vesicles derived from HEK293T cells	2017, J EXTRACELL VESICLES	ZHU XH	417	46.33
8	Exosome-delivered circRNA promotes glycolysis to induce chemoresistance through the miR-122-PKM2 axis in colorectal cancer	2020, MOL ONCOL	WANG XY	396	66.00
9	Cancer cell-derived exosomal circUHRF1 induces natural killer cell exhaustion and may cause resistance to anti-PD1 therapy in hepatocellular carcinoma	2020, Mol Cancer	Zhang PF	390	65.00
10	Tripodi M, De Leo G, Conigliaro Als modulate endothelial cell phenotype through the release of exosomes containing H19 lncRNA	2015, Mol Cancer	Conigliaro A	386	35.09

### 3.7 Citation spike analysis in references

**Figure 7:**
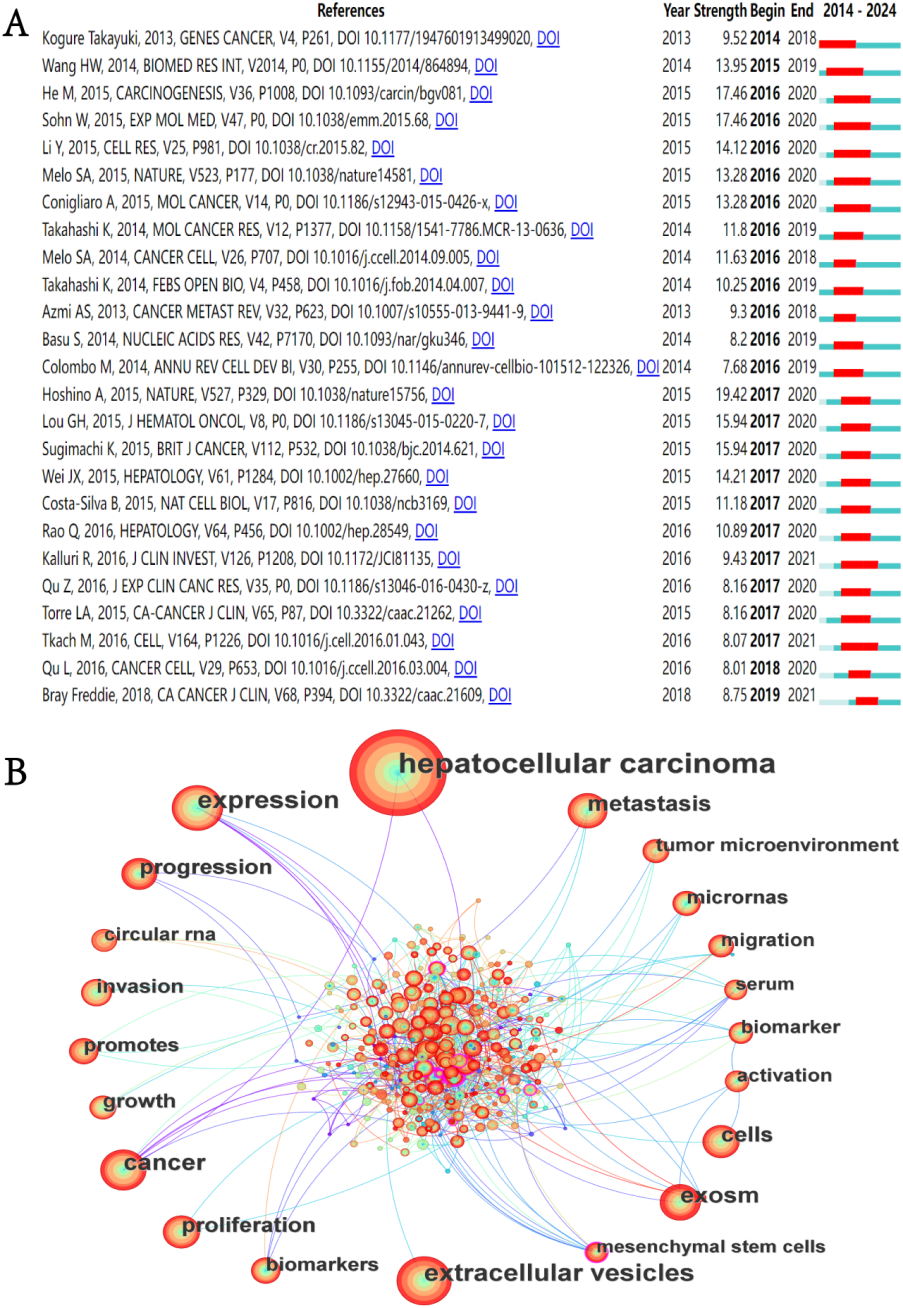
**(A)** Top 25 References with the highest citation bursts. **(B)** Keyword co-occurrence network diagram.

In the academic field, a citation explosion refers to the phenomenon where certain publications receive a substantial volume of citations within a brief timeframe, a volume significantly higher than the average citation count for literature in the same field. This phenomenon usually signals the formation of academic hotspots (30). This is because only those works that touch the academic frontier and put forward innovative ideas or methods may receive widespread attention and be cited in large numbers within a short period of time. In-depth analysis of these publications and provide useful guidance and serve as references for subsequent research. We utilized CiteSpace to identify the 25 references exhibiting the most significant citation bursts (**Figure 7A**). The temporal citation pattern is depicted by the blue line (2014-2024), whereas the red line highlights periods of citation bursts. The minimum observed burst duration was one year. The reference with the highest citation burst value is the article titled “Tumour exosome integrins determine organotropic metastasis” by Hoshino, A et al., published in Nature (2017–2020; citation burst=19.42) (31). The reference with the second-highest citation burst value is titled “HCC-derived exosomes promote motility of immortalized hepatocytes through transfer of oncogenic proteins and RNAs”, published in Carcinogenesis by He, M et al. in 2015 (32). The study found that exosomes derived from motile HCC cell lines can significantly enhance the migration and invasion capabilities of non-motile MIHA (immortalized hepatocytes), and activate the PI3K/AKT and MAPK signaling pathways in MIHA cells. This study is the first to confirm that exosomes secreted by HCC cells can induce the migration of immortalized hepatocytes, which may be related to enhancing the activity of HCC cells in traversing the liver parenchyma during metastasis.

### 3.8 Keyword analysis

Our systematic analysis identified 3930 keywords from the literature associated with the field of exosomes in HCC. **Figure 7B** shows the keyword co-occurrence network. The size of the nodes in the network represents. **Table 6** presents the 20 most prevalent keywords ranked by occurrence frequency. The top ten keywords, each appearing more than 80 times, are “hepatocellular carcinoma”, “extracellular vesicles”, “expression”, “cancer”, “exosomes”, “metastasis”, “cells”, “proliferation”, “progression”, and “growth”. Among these, “hepatocellular carcinoma” is the most frequently used, with 748 occurrences. This is followed by “extracellular vesicles” (254 occurrences) and “expression” (253 occurrences).

**Table 6:** The top 20 most frequently appearing keywords.

Rank	keyword	Frequency	Rank	Keyword	Frequency
1	hepatocellular carcinoma	748	11	invasion	80
2	extracellular vesicles	254	12	micrornas	75
3	expression	253	13	promoters	75
4	cancer	188	14	biomarkers	73
5	exosomes	160	15	tumor microenvironment	67
6	metastasis	141	16	migration	61
7	cells	130	17	activation	61
8	proliferation	118	18	circular rna	59
9	progression	110	19	mesenchymal stem cells	58
10	growth	82	20	serum	54

**Figure 8:**
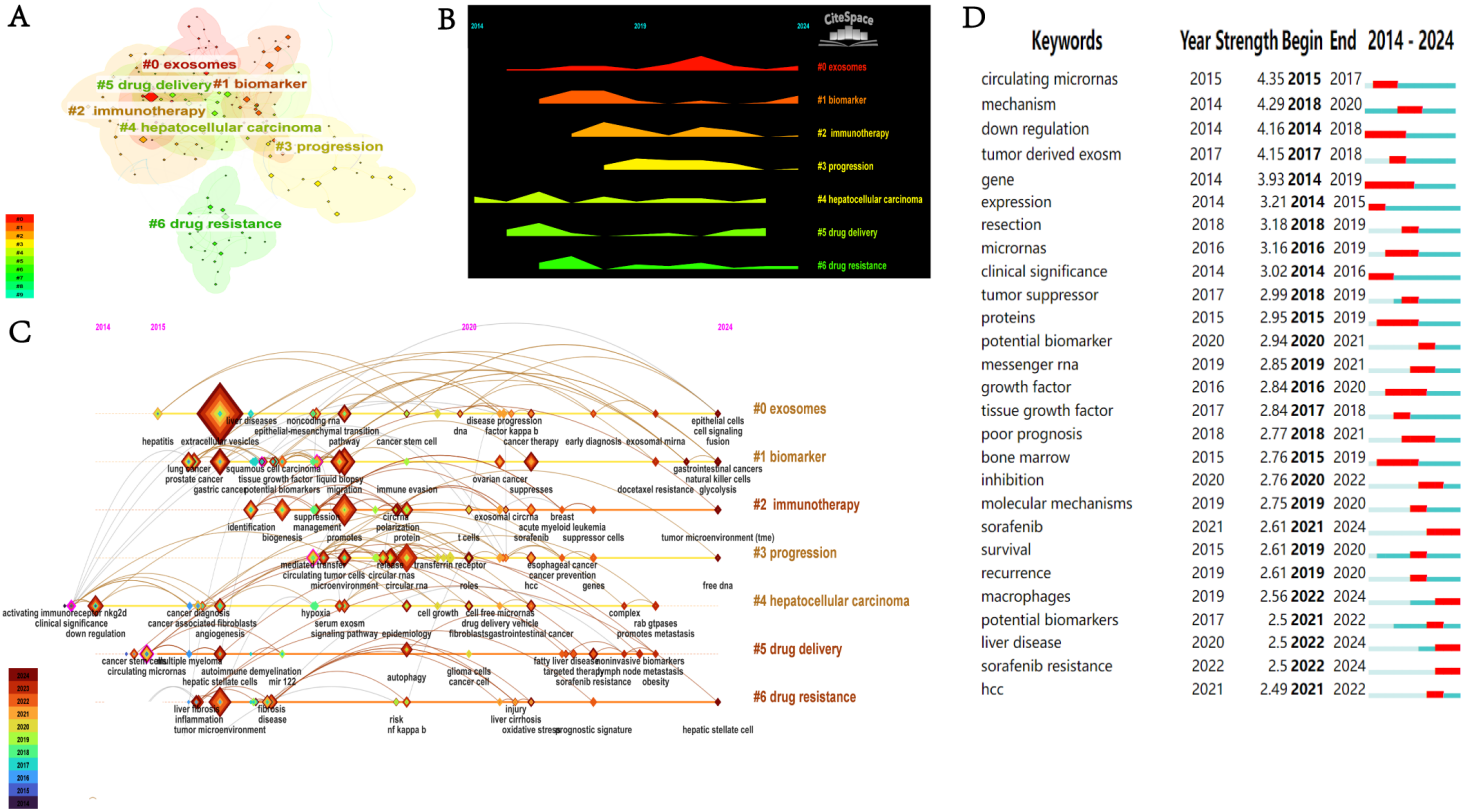
**(A)** Keyword co-occurrence clustering network. **(B)** Keyword landscape graph. **(C)** Keyword timeline map. **(D)** Top 27 keywords with the highest citation bursts.

To accurately identify the research themes and their evolution within this field, we conducted a clustering analysis of keywords. **Figure 8A** identified seven keyword clusters: #0 exosomes, #1 biomarker, #2 immunotherapy, #3 progression, #4 hepatocellular carcinoma, #5 drug delivery, and #6 drug resistance. Based on the results of the above cluster analysis, we created a (**Figure 8B**) and timeline view (**Figures 8C**). As indicated by the peak map and timeline graph, from 2014 to 2024, exosomes have evolved from an initially novel biomarker into a core tool for multi-dimensional interventions in diagnosis, treatment, and prognosis. Future focus will center on more complex mechanistic studies, as well as advancing the clinical validation of exosome-based drug carriers and drug resistance biomarkers.

**Figure 8D** shows the citation explosion of 27 keywords in the field of HCC exosomes. A citation burst is characterized by a significant and rapid increase in citation frequency of a specific keyword over a particular period, which usually reflects an emerging research hotspot or a significant new discovery within the field. The keywords with the highest intensity of citation bursts were “circulating miRNAs” (2015–2017, intensity=4.35), “mechanism” (2018–2020, intensity=4.29), and “down-regulation” (2014–2018, intensity=4.16). In addition, “sorafenib” (2021–2024), “macrophages” (2022–2024), and “sorafenib resistance” (2022–2024) have shown prominent recent bursts, indicating that they represent key research topics that have garnered substantial attention in recent years and are poised to remain prominent in the foreseeable future.

## 4 Discussion

### 4.1 Fundamental information

This study employs CiteSpace, VOSviewer, and R-based analytical tools in a comprehensive bibliometric approach to systematically evaluate the evolving landscape of HCC exosome research over the past decade. Through knowledge mapping visualization, we characterize international and institutional collaboration networks, identify emerging research themes, and delineate current research paradigms with their developmental trajectories. The resultant analytical framework provides both a state-of-the-art assessment of the field and forward-looking perspectives to guide future investigative priorities in exosome-based HCC research.

In academic evaluation metrics, a scholar’s publication output within a specific research domain is widely regarded as a quantifiable measure of their disciplinary engagement and prioritization (33). According to the publication trend, the trend line in **Figure 2** further confirms progressive growth in publications, highlighting that research on HCC-related exosomes is continuously expanding and garnering increasing attention. The high growth rate in recent years has been facilitated by the increased attention and support given to this field by a growing number of organizations. Between 2014 and 2024, a total of 1,120 publications related to the field of HCC exosomes were identified across 388 journals. The aforementioned journals are valuable resources for HCC exosome research. They provide a platform that supports both scientific inquiry and scholarly exchange in this field. International Journal of Molecular Sciences published the maximum publication volume and had the aggregate citation count and citation per paper rate, suggesting that this journal has the potential to become a prominent source for future research on exosomes in HCC therapy and more attention should be paid to this topic. Additionally, regarding citation impact, Molecular Cancer has published just 19 articles, yet it has accumulated an impressive total of 4,308 citations, resulting in an average of 226.74 citations per article. In comparison, Hepatology has released 12 articles, which received a total of 1,533 citations, averaging 127.75 citations per article. These statistics demonstrate a significant level of academic influence and imply that the quality of the published research is outstanding. Both journals are located in the UK and the US, respectively, with the investigation of exosomes in HCC emerging as a key research area. The elevated citation counts highlight the active research within this field and affirm the credibility of these journals. By analyzing the cooperation among countries, institutions and authors, we can not only present the quantity of articles clearly, but also visually reveal the connection between them, and also help researchers to utilize the available resources more efficiently, so as to provide references for the research in the field of HCC exosomes. In the field of HCC exosome research, China and the United States dominate. A geographic distribution map shows that most studies originate from China, the United States, and a handful of European countries; darker shading in the figure underscores their outsized scientific output. These nations combine strong economies with cutting-edge technology, and their pre-eminent research institutes, ample funding, and deep talent pools secure their influential and enduring presence. By contrast, participation from developing countries remains conspicuously low. Limited economic development and technological infrastructure restrict most of these nations’ budget allocations and capacity-building efforts, creating objective constraints on their research activities. Therefore, intensified international cooperation is essential: through shared resources and complementary strengths, developing countries can enhance their research capabilities and foster a more balanced global scientific landscape.

Given China’s substantial publication output, it is logical that the majority of the top 11 institutions are Chinese. Despite occupying the second-ranking position in terms of the quantity of publications, the United States does not boast an institution in the top 11. Fudan University and Zhejiang University have the highest number of publications and total citations, indicating that these two institutions have contributed the most to the field of exosomes in HCC and have the greatest impact in this field. These institutions are key contributors in the field, and their researchers have collaborated closely with many high-impact scholars. Zhang Q published research focusing on the molecular mechanisms of exosome genesis, immune escape, and metastasis in HCC, and elucidated the pivotal roles of non-coding RNAs and exosomes in the malignant behaviors of HCC. Patel T focused on non-coding RNAs in HCC, particularly exploring their biomarker potential and signaling pathway mechanisms in the disease. Zhou J, focusing on clinical translation, developed exosomes and their contents as diagnostic tools and therapeutic vectors, and advanced the translation of this research from mechanistic studies to clinical applications. In summary, although exosome research in HCC has been a popular topic among various countries, institutions, and authors, it is imperative that collaboration and communication between different institutions and research teams be enhanced. Moreover, analysis of highly cited references and citation bursts reveals that the field of HCC exosome research from 2014 to 2024 has established a knowledge system consisting of several high-impact core literatures, and experienced a research boom led by groundbreaking discoveries between 2015 and 2020. However, the number of papers with citation bursts has been relatively small in the past two years (2022-2024), suggesting that the field may be facing technical or resource bottlenecks. We believe that overcoming current bottlenecks, deepening clinical translation research, and exploring new interdisciplinary intersections will be key to revitalizing the field of HCC exosomes and generating the next generation of breakthroughs. The fundamental mechanisms established by early core work will remain an indispensable starting point for future research.

### 4.2 Research hotspots and trends

From the co-occurrence analysis results, the following keywords appeared with high frequency: “hepatocellular carcinoma”, “extracellular vesicles”, “expression”, “cancer”, “exosomes”, “metastasis”, “cells”, “proliferation”, “progression”, and “growth”. The clustering analysis categorized the keywords into seven clusters: #0 exosomes, #1 biomarker, #2 immunotherapy, #3 progression, #4 hepatocellular carcinoma, #5 drug delivery, and #6 drug resistance.

As revealed by the mountain plot (**Figure 8B**) and timeline view (**Figure 8C**), exosome research in the field of HCC has shown a clear and coherent evolutionary trajectory. Early studies mainly focused on the basic characteristics of exosomes themselves, such as exosomal miRNA and circular RNA, as well as the exploration of their potential as biomarkers. After 2017, research progressed to investigating complex mechanistic aspects, including their regulation of the tumor microenvironment, impact on disease progression, and drug resistance mechanisms, ultimately evolving toward applications with greater translational value, such as immunotherapy and their use as drug delivery vehicles to address clinical challenges. Although the themes of exosome-related research have continued to expand and deepen, hepatocellular carcinoma has remained the core focus across all clusters. Through cluster analyses of keywords, we have clearly delineated the five core themes in exosome research related to HCC and their evolutionary trends. This section will delve into these themes, integrating our reflections on the current state and future of the field.

#### 4.2.1 The role of exosomes in tumorigenesis, progression in HCC

HCC, the predominant type of primary liver cancer, continues to be a predominant cause of cancer-related mortality worldwide, largely due to its asymptomatic early stages, rapid progression, and limited therapeutic options (34). Recent studies highlight the tumor TME as a critical regulator of HCC malignancy, driving its aggressive evolution (35). The TME comprises diverse cellular components and non-cellular elements that collectively shape tumor behavior (36). A case in point is Feng et al., who evidenced that cancer-associated fibroblast (CAF)-derived exosomes deliver ZNF250 protein to HCC cells, upregulating PD-L1 expression to suppress CD8+ T cell activity while enhancing tumor proliferation, migration, and invasion (37). HCC-derived exosomes can also reprogram normal hepatocytes or neighboring tumor cells by activating migration-related signaling pathways (38). Mechanistically, these vesicles transfer bioactive molecules to recipient cells, triggering epithelial-mesenchymal transition (EMT) and fostering invasive phenotypes (39, 40).Notably, this EMT-inducing capability may partially explain the clinical phenomenon of early micro-metastasis in HCC. We hypothesize that targeting the exosome-mediated EMT signaling axis could serve as a novel strategy to intervene in micro-metastasis, though further validation of its specificity is needed to avoid damage to normal tissues.

Furthermore, exosome-mediated crosstalk between HCC cells and non-immune TME components has emerged as a key research focus. HCC exosomes carrying HMGB1 drive the expansion of TIM-1+ regulatory B cells, facilitating immune evasion (41). Similarly, exosomal lncRNA TUC339 impairs natural killer (NK) cell cytotoxicity, further compromising antitumor immunity (42).In vascular regulation, Cao et al. revealed that exosomal miR-21 activates VEGF signaling in CAFs by targeting TIMP3 mRNA, promoting pathological angiogenesis (43). Notably, most of these studies focus on a single molecule or pathway. The heterogeneity of HCC means that exosome-mediated TME remodeling is a highly complex networked event. Future research should integrate multi-omics approaches to more systematically map the overall role of exosomes in constructing a tumor-promoting microenvironment and identify key “hub” molecules. Deng et al. demonstrated that E2F1 transcriptionally up-regulates EXOSC10 to augment exosome secretion, thereby driving HCC stemness and proliferation (44), uncovering a novel mechanism by which a transcription factor modulates exosome biogenesis to sculpt tumor traits. From this, we hypothesize that targeting the critical enzymes or master regulators of exosome biogenesis may yield broader therapeutic benefits than interfering with individual exosomal cargos. For instance, the small-molecule GW4869 can block exosome production in multiple preclinical models (45); however, its *in vivo* anti-metastatic efficacy, long-term safety, and synergistic potential with current therapies—such as sorafenib or immune checkpoint inhibitors—require rigorous evaluation. Selective inhibition of exosome generation in specific cellular sources could therefore minimize off-target liabilities. Encouragingly, the tumor-promoting effects mediated by exosomes are reversible. Chen et al. successfully reversed the migration, invasion, and EMT phenotypes of HCC cells using engineered exosomes loaded with miR-1246 inhibitors (46). This provides proof-of-concept for exosome-based “corrective” therapies. However, challenges lie in efficiently and specifically delivering therapeutic molecules to target tumor cells or TME cells, as well as overcoming *in vivo* barriers. The stability and large-scale production of engineered exosome drug-loading systems represent key bottlenecks for clinical translation.

#### 4.2.2 Exosomes as early diagnostic and prognosis biomarkers for HCC

The early diagnosis of HCC faces significant challenges, as the covert clinical symptoms lead to approximately 70% of patients being diagnosed at an advanced stage. Although the current clinical standards rely on alpha-fetoprotein (AFP) testing, imaging assessments, and histopathological examinations, these methods have evident limitations (47–49). In recent years, exosome liquid biopsy technology has demonstrated breakthrough diagnostic potential due to its advantages of multi-source nature (such as blood, urine), integrity of molecular information, and high stability.

In terms of molecular mechanism research, HBV-related HCC patients exhibit characteristic changes in the miRNA expression profile of exosomes, such as significant downregulation of miR-18a and miR-221 (50). However, the sensitivity limitations of traditional detection methods and their equipment dependency have constrained clinical translation. The PERFECT technology developed by the Lai team has achieved a revolutionary breakthrough. This technology overcomes conventional temperature limitations and simplifies the detection process, providing a new type of molecular diagnostic tool for point-of-care testing of exosomal miRNA biomarkers. A multi-omics joint diagnostic strategy further enhances detection efficiency (51). However, this technology still faces challenges in distinguishing tumor-derived exosome subpopulations. We believe that future efforts should integrate real-time capture technologies targeting exosomal surface-specific markers to enhance the precision of selective detection for HCC-derived exosomes. The combined detection of miRNA-122 and lncRNA can improve diagnostic performance (52). Studies have shown that exosomal circRNAs, as novel biomarkers, not only significantly enhance clinical diagnostic efficiency when combined with traditional markers but also provide precise and personalized treatment options for HCC patients (53). Zhang et al. further proposed that exosomal circRNAs could function as biomarkers for early diagnosis and prognosis assessment in cases of HCC (54). Detectable across multiple biofluids, these molecules exhibit high abundance, stability, and sensitivity, allowing minimally invasive sampling, and may represent novel therapeutic targets to improve patient outcomes. We observe that circRNA research is rapidly emerging, but its specificity and functional mechanisms within HCC exosomes, along with rigorous large-scale clinical validation, remain insufficient. More prospective cohort studies are needed to rigorously evaluate its diagnostic utility and clinical application value. The introduction of AI (such as the ChatExosome system) provides a powerful tool for interpreting complex exosomal multi-omics data (55). However, its effectiveness is dependent on access to large volumes of accurately annotated, high-quality data.

Overall, exosome liquid biopsy represents a promising avenue for the early diagnosis of HCC. However, its transition into routine clinical practice faces multiple obstacles: the lack of standardized isolation methods, balancing isolation efficiency with cost, the rigor required for biomarker validation, and whether its sensitivity and specificity can meet the demands of clinical screening. Establishing industry consensus and standardized protocols is imperative.

#### 4.2.3 The role of exosomes in HCC drug resistance

According to the most recent studies on the topic, exosome function has been demonstrated to play a key regulatory role in the context of treatment resistance in tumors (56). HCC, as a typical highly drug-resistant tumor, poses a severe challenge to treatment. Sorafenib has been established as the initial systemic therapeutic approach for cases of advanced HCC, and frequently encounters resistance that severely limits clinical outcomes. Current research has found that exosomes mediate HCC resistance through various mechanisms. For example, exosomes can mediate HCC resistance through nucleic acid molecular regulatory mechanisms. circRNA-SORE significantly enhances HCC cell resistance to sorafenib by activating the YAP signaling pathway (57). However, further research is needed to determine the clinical value of targeting circRNA-SORE to overcome sorafenib resistance in HCC patients. Li W et al. identified exosomal miR-6126 as a key regulator of sorafenib resistance in HCC (58).Using *in vitro* models of sorafenib-resistant Huh7 cells combined with exosome isolation and multi-omics analysis, they observed 180 differentially expressed exosomal miRNAs, with miR-6126 being significantly downregulated in resistant cells. This miRNA reversed drug resistance by modulating CD44/HK2 expression and intracellular pH dynamics, highlighting its potential as a novel therapeutic target to overcome HCC treatment resistance. Additionally, exosomal miR-32-5p induces a multidrug-resistant phenotype through the PTEN/PI3K/Akt pathway, and lncRNA regulatory networks are involved in the formation of resistance (59). Exosomes can also mediate HCC resistance by regulating the tumor microenvironment. In a groundbreaking study, Jing C et al. first discovered and confirmed that exosomes derived from M2-type macrophages can promote HCC resistance to sorafenib (60). This finding offers a new perspective and potential intervention target for the clinical treatment of HCC (61). In addition, tumor-derived exosomes can further enhance tumor cell resistance by inducing apoptosis (62–64).

In summary, although current research has revealed multiple exosome-mediated drug resistance mechanisms, the following key issues remain to be addressed: Drug resistance in HCC often stems from the synergistic effect of multiple exosomal pathways, and the efficacy of single-target intervention is limited. There is an urgent need to identify more upstream core regulatory factors or exosome biogenesis nodes that can coordinate multiple drug resistance pathways; Most existing mechanisms are based on cell models, which differ significantly from the complex *in vivo* microenvironment. The effectiveness and safety of factors such as circRNA-SORE and miR-6126 still need to be systematically verified in preclinical animal models and early clinical trials; The specificity of exosome biomarkers related to drug resistance is insufficient and they are susceptible to interference. Moreover, as a component of liquid biopsy, the potential of exosomes in real-time monitoring of drug resistance occurrence during treatment and evaluating the efficacy of reversal strategies has not been fully explored. With the continuous development of cancer treatment methods, the problem of drug resistance has become increasingly prominent, and challenges such as insufficient specificity of drug-resistant exosome biomarkers are particularly significant. Therefore, when exploring exosome-targeted therapies for HCC, researchers must be highly vigilant about potential drug resistance risks and promote clinical translation by establishing industry consensus and standardized procedures.

#### 4.2.4 Exosome-based drug delivery

Studies have confirmed that exosomes, as drug delivery carriers, possess significant advantages such as precise targeting of tumor cells and reduction of side effects (64, 65) making them a research hotspot in this field. However, their clinical application still faces key challenges: exosomes are prone to degradation and aggregation during isolation, storage, and administration, which affects their stability, and inter-/intratumoral heterogeneity in HCC leads to therapeutic resistance. Emerging bioengineering approaches, through the rational design of vesicle platforms, are expected to achieve precise RNA delivery and patient-specific treatments. Nevertheless, engineered exosomes still encounter severe challenges in HCC applications, including efficient and stable drug loading with targeted release, technical bottlenecks and high costs in large-scale production and quality control, as well as the lack of sensitive and non-invasive *in vivo* tracking technologies.

We believe that the key to addressing these challenges lies in interdisciplinary technological innovation. In terms of advanced drug loading strategies, more gentle and efficient loading technologies (such as microfluidics and sonoporation) can be developed, or ‘smart’ responsive drug delivery systems can be designed by leveraging the membrane fusion properties of exosomes. For precise targeting, the ability to actively target HCC cells or specific TME components can be conferred through genetic engineering modification of parent cells or chemical/biological modification of exosome membranes. Additionally, bionics can be combined with synthetic biology to design synthetic nanovesicles by drawing on the natural components of exosomes. Meanwhile, it also crucial to develop high-sensitivity molecular imaging technologies or detection methods based on the intrinsic signals of exosomes for *in vivo* tracking, as well as other advanced characterization and tracking technologies.

Preclinical studies have shown that the use of engineered exosomes to deliver chemotherapeutic agents, RNAi drugs, etc., exhibits the potential to enhance efficacy and reduce toxicity in various tumor models (66, 67) and these findings lay a foundation for their application in HCC. Furthermore, recent advancements in isolation and purification technologies have greatly expanded the source options for exosomes. Current production platforms utilize both *in vitro* cell culture systems and endogenous biological sources, providing multiple scalable production pathways to meet the growing therapeutic demands. This diversification of production methods facilitates the transition to large-scale clinical applications. Clinical validation of exosome-based therapies continues to accumulate across multiple disease indications.

#### 4.2.5 Exosome-based immunotherapy

Exosomes play a crucial role in immunotherapy for HCC. As multifunctional communication hubs within the TME, they regulate immune responses through multiple mechanisms, including presenting tumor antigens, delivering immunomodulatory molecules, and directly interacting with immune cells (68). This underscores the significance of exosomes as versatile communication hubs in the TME, making them ideal targets and carriers for the development of immunotherapies. Studies have shown that the TME significantly promotes immune evasion in HCC by releasing immunosuppressive factors and activating related pathways (69). This research deepens our understanding of the complexity of immunosuppression in HCC, suggesting that targeting TME-derived exosomes or their carried inhibitory factors is one of the key strategies to overcome immune evasion. Furthermore, existing studies have confirmed that exosomes exert regulatory effects in immune responses, among which tumor-derived exosomes (TDE/TEX) are key carriers that perform immunosuppressive functions. Research indicates that TDEs possess distinct immunosuppressive activity and can promote tumor growth (70); meanwhile, as important immunomodulators in the TME, TEXs mediate interactions between tumor cells and various immune cells, thereby facilitating immune evasion and tumor progression (71). In terms of therapeutic applications, Zuo B et al. proposed a bio-nano vaccine strategy based on universal dendritic cell-derived exosomes (DEXs) (72). This study confirmed that such DEX vaccines can trigger personalized tumor-specific immune responses tailored to individual HCC tumors, providing a promising and universal approach for the development of personalized HCC immunotherapies.

However, despite the great potential of exosomes in HCC immunotherapy, their clinical application still faces significant challenges: it is urgent to achieve standardization and scaling of exosome production, optimize efficient and targeted loading technologies for therapeutic molecules, and rigorously verify their clinical safety and efficacy. A thorough understanding of the *in vivo* distribution, clearance mechanisms, and potential toxicity of exosomes is a prerequisite for ensuring their clinical safety. Successfully overcoming these obstacles will open up broad prospects for exosome-based immunotherapy in HCC. In conclusion, future research should adopt a strategy of interdisciplinary integration. On the basis of gaining a deep understanding of the biology of HCC exosomes, it is essential to focus on breaking through the technical bottlenecks in the production and loading of engineered exosomes, systematically evaluate their safety and efficacy, and promote this promising therapeutic strategy from the laboratory to the clinic through well-designed clinical trials, ultimately benefiting HCC patients.

### 4.3 Prospects and challenges

Research on exosomes in HCC shows great potential but also faces significant challenges. In the field of diagnosis and monitoring, integrating multi-omics information such as exosome-derived circRNA and miRNA with artificial intelligence algorithms is expected to enable non-invasive diagnosis of HCC and real-time dynamic monitoring of therapeutic efficacy and drug resistance. It is anticipated that exosome-based liquid biopsy protocols will be incorporated into clinical guidelines within the next five years.

In terms of overcoming therapeutic resistance, in-depth analysis of key exosome-mediated mechanisms will drive the development of new targeted drugs or strategies, effectively reversing resistance to first-line drugs such as sorafenib. For therapeutic applications, engineered exosomes, as revolutionary multifunctional delivery platforms, have significant advantages in efficiently loading chemotherapeutic drugs, nucleic acid drugs, and immunomodulators. More such therapies will enter clinical trials in the next five years, and their combination with existing standard treatments (immunotherapy, local therapy) will become mainstream. Of particular note is the use of engineered exosomes to deliver immunomodulators, which can reshape the tumor microenvironment and enhance the sensitivity to immunotherapy.

However, the development of this field is still constrained by multiple severe challenges. The primary challenge stems from the high heterogeneity of HCC, which makes it extremely difficult to identify universal biomarkers and therapeutic targets. Additionally, the cross-omics integration analysis of exosomal non-coding RNA, proteome, and HCC genome data remains insufficient, hindering the development of biomarkers with high specificity and sensitivity. The clinical translation of engineered exosomes faces bottlenecks such as drug loading and targeting efficiency, *in vivo* tracking and evaluation systems, and quality control in large-scale production; standardized methods for exosome isolation are also lacking. Furthermore, as a new type of biological agent, its regulatory framework is still being improved, and the high R&D and production costs may affect clinical accessibility. Looking forward, efforts should be made to develop engineered exosome platforms specifically targeting HCC and explore their value in delivering new therapies and combination treatments. Through cross-omics analysis methods, highly specific and sensitive biomarkers should be developed to promote the clinical application of exosomes in immunotherapy, drug resistance, and drug delivery.

It should be noted that this study, as a bibliometric analysis, relies on specific software workflows and algorithms. Although it efficiently reveals macro trends and knowledge structures, it cannot fully replace systematic manual in-depth literature reading and content analysis. The data are mainly derived from English literature in the Web of Science Core Collection, posing a risk of missing important non-English research findings. Moreover, given the rapid development of exosome research, some of the latest breakthroughs within the analysis time window may not have been fully cited or reflected. Nevertheless, this study, through a combination of quantitative analysis and qualitative trend interpretation, provides researchers with a systematic overview of the development context, core focuses, knowledge foundations, and emerging frontiers in the field of HCC exosomes over the past decade, which will be beneficial for future research in this area.

## 5 Conclusion

Our bibliometric analysis reveals that research on exosomes in HCC is attracting growing attention within the research community. Over the past decade, the research focus has shifted from the fundamental characterization of exosomes toward their clinical applications and mechanistic studies, directions which demonstrate significant translational potential. Current research trends indicate several promising future avenues for exploration: developing and validating novel diagnostic biomarkers derived from exosome cargo; optimizing exosome-based drug delivery systems for targeted therapy; investigating the role of exosomes in HCC drug resistance; and exploring exosome-mediated immunotherapeutic strategies for HCC to facilitate their clinical application. In summary, this study represents the first comprehensive bibliometric analysis of the literature in the field of HCC exosomes. Through a systematic examination of the field’s developmental trends and key research themes, these findings provide researchers with reliable guidance to identify emerging patterns and hotspots, offering valuable insights for designing future innovative research in this domain.
